# MPTP-Induced Dopamine Depletion in Basolateral Amygdala *via* Decrease of D2R Activation Suppresses GABA_A_ Receptors Expression and LTD Induction Leading to Anxiety-Like Behaviors

**DOI:** 10.3389/fnmol.2017.00247

**Published:** 2017-08-07

**Authors:** Tingting Zhang, Tingting Chen, Peipei Chen, Baofeng Zhang, Juan Hong, Ling Chen

**Affiliations:** ^1^State Key Lab of Reproductive Medicine, Nanjing Medical University Nanjing, China; ^2^Department of Physiology, Nanjing Medical University Nanjing, China

**Keywords:** dopaminergic receptor (DR), anxiety-like behaviors, basolateral amygdala (BLA), synaptic plasticity, GABA_A_ receptor (GABA_A_R)

## Abstract

Anxiety disorders commonly occur in Parkinson’s disease. Using field potential recording and patch-clamp recording, we evaluated influence of MPTP-reduced dopaminergic afferent in basolateral amygdala (BLA), a main region for affective regulation, on excitatory–inhibitory circuits and synaptic plasticity. Field excitatory post-synaptic potential (fEPSP) slopes at external capsule-BLA synapses were increased in MPTP-mice with decreases in paired-pulse facilitation and long-term potentiation amplitude, which were corrected by bath-application of D2R agonist quinpirole or cannabinoid type 1 receptors agonist WIN55,212-2, but not D1R agonist SKF38393. Compared to single waveform fEPSP in control mice, a multi-spike waveform fEPSP was observed in MPTP-mice with prolongation of duration and an increase in paired-pulse inhibition, which were recovered by BLA-injection of quinpirole for 2 days rather than bath-application. Density of GABA-evoked current (*I*_GABA_) in BLA principal neurons and GABA_A_R-α2 subunit expression were reduced in MPTP-mice, which were recovered by administration of quinpirole. Decline of PKC phosphorylation in BLA of MPTP-mice was corrected by bath-application of quinpirole, but not SKF38393. In MPTP-mice, BLA-injection of quinpirole or PKC activator PMA could recover GABA_A_R expression, which was sensitive to PKC inhibitor GF109203X. The impairment of long-term depression (LTD) in MPTP-mice was rescued by bath-application of GABA_A_R agonist muscimol or BLA-injection of quinpirole and PMA. Finally, BLA-injection of muscimol, quinpirole or PMA relieved anxiety-like behaviors in MPTP-mice. The results indicate that the MPTP-induced dopamine depletion in BLA principal neurons through reducing D2R-mediated PKC phosphorylation suppresses GABA_A_R expression and activity, which impairs GABA_A_R-mediated inhibition and LTD induction leading to anxiety-like behaviors.

## Introduction

Parkinson’s disease (PD) is a neurodegenerative disorder characterized by motor symptoms and a progressive loss of dopaminergic neurons (Braak et al., 2003; Rodriguez-Oroz et al., 2009). Anxiety disorders and depression commonly occur in PD patients, affecting up to 40% of patients at early stages of the disease (Walsh and Bennett, 2001). L-DOPA therapy can improve anxiety disorders in PD (Shin et al., 2014), suggesting that the affective disorders may be linked to a dopamine deficiency.

The basolateral nucleus of the amygdala (BLA), a main region for affective regulation, receives dense dopaminergic innervations from the ventral tegmental area (VTA). Using dual-labeling immunohistochemistry, Muller et al. (2009) have demonstrated that the principal neurons are the primary targets of the dopaminergic inputs to the BLA. The BLA principal neurons express the D1-like receptor (D1R) and D2-like receptor (D2R) (Pinto and Sesack, 2008). The activation of D1R increases excitability of BLA principal neurons, whereas the activation of D2R increases input resistance (Kroner et al., 2005). The activation of D2R induces the release of post-synaptic endocannabinoids, which act as retrograde messengers *via* activation of cannabinoid type 1 receptors (CB1R) to inhibit glutamate release at cortico-striatal synapses (Yin and Lovinger, 2006). A large body of evidence indicates that hyperexcitability of the BLA principal neurons is associated with affective disorder in rodents and humans (Senn et al., 2014; Prager et al., 2016). The fear memory formation requires the induction of long-term potentiation (LTP) in the BLA principal neurons (Goosens and Maren, 2002), while long-term depression (LTD) is thought to facilitate the extinction of learned fear (Dalton et al., 2012). Recently, the impairment of LTD induction in BLA has been reported to cause the production of depressive-like behaviors (Zhang et al., 2017). By modulating the activity of amygdala neurons, dopaminergic neurons are known to control the expression of fear memory (Fadok et al., 2009; de la Mora et al., 2010). However, the influence of dopaminergic deficiency on the synaptic plasticity in BLA and affective behaviors remain to be elucidated.

The interaction between the dopaminergic, GABAergic, and glutamatergic inputs represents an important aspect of the functional regulation in BLA (Soghomonian and Chesselet, 2000). BLA output is controlled by a local feedback inhibition of GABAergic interneurons (Diaz et al., 2011). GABAergic interneurons can lead to a low resting firing rate of principal neurons (Woodruff and Sah, 2007). GABAergic disruption results in hyperexcitability of BLA principal neurons (Prager et al., 2016). The microinjection of GABA_A_ receptors (GABA_A_R) agonists and antagonists in amygdala can exert anxiolytic or anxiogenic-like effects, respectively (Barbalho et al., 2009). Dopamine is reported to control acquisition of fear memory by increasing inhibitory post-synaptic currents (IPSCs) in the lateral amygdala (Rosenkranz and Grace, 2002; Bissière et al., 2003).

The administration of neurotoxin 1-methyl-4-phenyl-1,2,3,6-tetrahydropyridine (MPTP) in C57BL/6 mice reduces the dopaminergic fibers within the BLA (von Bohlen und Halbach et al., 2005) and causes the affective disorders (Schober, 2004). The expression of GABA_A_R in the rostral globus pallidus is decreased in MPTP-treated monkeys (Calon et al., 1995). The aim of the present study was to investigate the influence of the MPTP-induced dopamine depletion in BLA on D1R and D2R-mediated excitatory–inhibitory circuits, synaptic plasticity, and GABA_A_R activity and expression. We further explored the association between the MPTP-induced dopamine depletion in BLA and production of affective disorders. Our results indicate that the MPTP-induced dopamine depletion in BLA through reducing D2R activation can suppress the GABA_A_R expression and function, which causes the deficits in the GABA_A_R-mediated inhibitory circuit and LTD induction leading to the production of anxiety-like behaviors.

## Materials and Methods

### Animal Preparation

All animal experiments were approved by Ethical Committee of the Nanjing Medical University and were performed in accordance with experimental animals guidelines established by the Laboratory Animal Research Institute. Eight-week-old (24–26 g) and 4-week-old (10–12 g) male C57BL/6 mice (Oriental Bio Service, Inc., Nanjing) were used at the beginning of the experiment. The mice were maintained under constant environmental conditions (temperature 23 ± 2°C, humidity 55 ± 5% and 12:12 h light/dark cycle) in Animal Research Center of Nanjing Medical University with free access to food and water. The mice received daily intraperitoneal (i.p.) injection of MPTP (25 mg/kg, measured as free base; Sigma-Aldrich, St. Louis, MO, United States) for 5 consecutive days (Crocker et al., 2003).

### Preparation and Administration of Drugs

D1R antagonist SCH23390, D2R agonist quinpirole, AMPA receptor antagonist CNQX, GABA_A_R agonist muscimol and GABA_A_R antagonist bicuculline, D2R antagonist L-sulpiride, PKA inhibitor H89 and PKC inhibitor GF109203X were purchased from Sigma-Aldrich, D1R agonist SKF38393 was purchased from Tocris, and PKC activator PMA, CB1R antagonist AM251 and CB1R agonist WIN55,212-2 were obtained from Medchemexpress. SKF38393, SCH23390, quinpirole, CNQX, muscimol, and bicuculline were dissolved in distilled water. L-sulpiride, H89, GF109203X and PMA, AM251 and WIN55,212-2 were dissolved in 1.0% DMSO for stock solutions.

For the bath-application of the slices, SKF38393 (10 μM), SCH23390 (10 μM), quinpirole (10 μM), L-sulpiride (20 μM), CNQX (10 μM), muscimol (10 μM), bicuculline (10 μM), AM251 (10 μM), and WIN55,212-2 (10 μM) were diluted by ACSF to a concentration of 0.1% DMSO (Bissière et al., 2003; Dallerac et al., 2011; Soni et al., 2014; Zhang et al., 2017). For *in vivo* administration, SKF38393 (10 mg/kg) and quinpirole (2 mg/kg) were intraperitoneally (i.p.) injected (Zhang et al., 2016). For micro-injection of BLA, mice were anesthetized with chloral hydrate (400 mg/kg, i.p.) and were then placed into a stereotaxic instrument (Stoelting, Wood Dale, IL, United States). A small hole (2 mm diameter) was drilled in the skull using a dental drill. A guide cannula (26-gauge, Plastics One, Roanoke, VA, United States) was implanted into the BLA (1.4 mm posterior, ± 3.0 mm lateral, and 4.8 mm ventral to bregma). On day 3 after surgery, the dummy cannula was removed from the guide cannula, and then replaced by infusion cannulas (30 gauge) connected by polyethylene tubing (PE10; Becton Dickinson, Sparks, MD, United States) with a stepper-motorized micro-syringe (Stoelting, Wood Dale, IL, United States). SKF38393 (0.1 μg/mouse), SCH23390 (0.25 μg/mouse), quinpirole (0.5 μg/mouse), L-sulpiride (0.25 μg/mouse), muscimol (4 nmol/mouse), H89 (2 nmol/mouse), PMA (48 pmol/mouse), and GF109203X (5 ng/side) were diluted with sterile saline and injected in a volume of 0.25 μl/side BLA (Kim et al., 2000; Zeni et al., 2012; Nasehi et al., 2016). Control mice were given an equal volume of vehicle. After 2% Evans Blue (0.5 μl) was injected, the mice were killed by an overdose of chloral hydrate, and coronal sections (100 μm) were cut using a cryostat to validate the injection site.

### Behavioral Examination

#### Open-Field Test

Mice were examined in a cuboid plexiglass box (60 cm × 60 cm × 40 cm) with the gray floor divided into 16 equal squares. The central zone was defined as the central 4 squares (30 cm × 30 cm). Boxes were evenly illuminated with white light (25 lx). Each mouse was placed in a corner of the arena and allowed to freely explore for 5 min. The following parameters were evaluated: (i) total distance traveled (mm/5 min), calculated as the number of partitions crossed within 5 min; and (ii) the time spent in the center region of the arena (Ping et al., 2014).

#### Elevated Plus-Maze

The apparatus consisted of two open arms (23.5 cm × 8 cm), painted white, and two enclosed arms (23.5 cm × 8 cm × 20 cm high), painted black. The maze was raised to a height of 38.5 cm above the floor. Lighting in the maze was 15 lx. Mice were placed in the center area facing one of the open arms and their movement and time spent in the different arms were analyzed for 5 min. The data were expressed as the time spent in the open arms (Zhang et al., 2010).

### Electrophysiological Analysis

#### Field Potential Recording

Mice (8-weeks-old) were decapitated under deep anesthesia with isoflurane. The brains were rapidly removed. The coronal slices (400 μm) were cut using a vibrating microtome (Microslicer DTK 1500, Dousaka EM, Co., Kyoto, Japan) in ice-cold oxygenated (95% O_2_/5% CO_2_) cutting solution composed of (in mM) 94 sucrose, 30 NaCl, 4.5 KCl, 1.0 MgCl_2_, 26 NaHCO_3_, 1.2 NaH_2_PO_4_, and 10 D-glucose, pH 7.4. After a 1-h recovery, the slices containing the cortex and amygdala were transferred to a recording chamber and continuously perfused with oxygenated ACSF composed of (in mM) 124 NaCl, 2 CaCl_2_, 4.5 KCl, 1 MgCl_2_, 26 NaHCO_3_, 1.2 NaH_2_PO_4_, and 10 D-glucose (pH value of ACSF was adjusted to 7.4) at 30 ± 1°C. A bipolar tungsten electrode was placed outside of the BLA to stimulate the external capsule (EC) fibers from the cortex. The constant current pulses (pulse width: 100 ms; frequency: 0.05 Hz) were supplied by a stimulator (SEN-3301, Nihon Kohden, Japan). Field excitatory post-synaptic potential (fEPSP) were recorded from the BLA with a 5-MΩ resistance glass microelectrode that was filled with 2 M NaCl and connected to a neutralized, high input-impedance preamplifier with a high-pass filter at 5 kHz. Signals were amplified with a differential AC amplifier (A-M Systems, model 1700, Seattle, WA, United States) and were digitized using the pCLAMP system (Axon Instrument, Inc., Sunnyvale, CA, United States). The test stimulus was set at approximately 50% of the maximum stimulus intensity that evoked a saturated fEPSP slope in each slice. Paired-pulse facilitation (PPF) or inhibition (PPI) was induced by paired-pulse stimulation with interpulse interval (IPI) of 75 or 25 ms, respectively. The paired-pulse ratio (PPR) was calculated with the following formula: (fEPSP_S2_/fEPSP_S1_) × 100, where fEPSP_S1_ and fEPSP_S2_ represent the fEPSP slopes evoked by the first and second stimulation, respectively. LTP was induced using high-frequency stimulation (HFS, 100 Hz for 1 s) consisting of five trains with 20 s intervals (Zhang et al., 2017). LTD was induced by low-frequency stimulation (LFS, 1 Hz for 15 min). The degree of LTP or LTD was expressed as a percent increase or decrease comparised with the baseline of the fEPSP slopes. The 20% greater or lower values of fEPSP slopes at 55–60 min after delivering HFS or LFS than baseline were considered as the induction of LTP or LTD.

#### Whole Cell Patch-Clamp Recording

Mice (4-week-old) were anesthetized with isoflurane and decapitated. The coronal section (400 μm) was cut using a vibrating microtome in ice-cold ACSF (in mM: NaCl 126, CaCl_2_ 1, KCl 2.5, MgCl_2_ 1, NaHCO_3_ 26, KH_2_PO_4_ 1.25, and D-glucose 20, pH 7.4) oxygenated with 95% O_2_/5% CO_2_. The slices were incubated at 32–34°C using an in-line heating device (Warner Instruments, Hamden, CT, United States) for 60 min, and then transferred to a recording chamber. A glass pipette (4–5 MΩ resistance) was filled with an internal solution (in mM: CsCl 140, CaCl_2_ 1, MgCl_2_ 2, Tris-ATP 2, HEPES 10, EGTA 10) at pH 7.2. The slice was perfused continually using ACSF supplemented with bicuculline (10 mM), NBQX (10 mM), and TTX (0.1 mM). The holding potential was -60 mV. GABA-activated current (*I*_GABA_) in BLA principal neurons was induced by the application of GABA (1–300 μM) using a rapid drug delivery system (Hong Z. et al., 2016) and recorded using an EPC-10 amplifier (HEKA Elektronik, Lambrecht/Pfalz, Germany). The density of *I*_GABA_ was normalized to the maximum *I*_GABA_ recorded in the same neuron to produce a dose-response curve. The data were fitted to logistic equation in which *I* = *I_max_*/[1+(EC50/*C*)*n*], with *n* being the Hill coefficient and EC50 being the concentration producing a 50% maximal response.

### Western Blotting Analysis

The coronal sections (500 μm) from -1.5 mm to -2.0 mm relative to Bregma were cut using a cryostat microtome (CM1900, Wetzlar, Hessen, Germany) according to the mice brain atlas (Paxinos and Franklin, 2004). The region containing the BLA was harvested using a 15-gauge needle (inner diameter 1.5 mm) and homogenized in a lysis buffer containing 50 mM Tris-HCl (pH 7.5), 150 mM NaCl, 5 mM EDTA, 10 mM NaF, 1 mM sodium orthovanadate, 1% Triton X-100, 0.5% sodium deoxycholate, 1 mM phenylmethylsulfonyl fluoride and protease inhibitor cocktail (Roche, Germany). Protein concentration was determined with the BCA Protein Assay Kit (Pierce Biotechnology, Inc., United States). Total protein (20–50 μg) was separated by SDS-polyacrylamide gel electrophoresis and transferred to a polyphorylated difluoride membrane. The membranes were incubated with 5% non-fat dried milk for 60 min and then incubated in the antibodies of anti-GABA_A_R-α2 (1:500, Bioworld Technology), anti-PKA phosphorylation (1:1000, Cell Signaling Technology, Inc., Boston, MA, United States), anti-PKC phosphorylation (1:1000, Abcam, Cambridge, United Kingdom). The membranes were incubated at 4°C overnight with HRP-labeled secondary antibodies and developed using the ECL detection Kit (Millipore, Billerica, MA, United States). Following visualization, the blots were stripped by incubation in stripping buffer (Restore; Pierce) for 15 min; and then incubated with antibodies of anti-PKA (1:1000, Cell Signaling Technology), anti-PKC (1:1000, Abcam), anti-β-actin (1:1000, Cell Signaling Technology). BLA samples from six mice were repeated in triplicate. Western blotting bands were scanned and analyzed using the Image J analysis software package (NIH). The densitometric value of phosphorylated protein normalized by total protein was normalized by the controls.

### Reverse Transcription-Polymerase Chain Reaction (RT-PCR)

Total RNA was isolated from the BLA with TRIzol reagent (Invitrogen, Camarillo, CA, United States) and reverse-transcribed into cDNA using a Prime Script RT reagent kit (Takara, China) for quantitative PCR (ABI Step One Plus, Foster City, CA, United States) in the presence of a fluorescent dye (SYBR Green I; Takara, China). The relative expression of genes was determined using the 2^-ΔΔct^ method with normalization to *GAPDH* expression. The primer sets used for *D1R*, *D2R*, *GABA_A_R-α2*, *GABA_A_R-α4*, *GluR1*, *GluR2*, *NR2A,* and *NR2B* were designed according to the publications (Kim et al., 2010, 2015; Zhou et al., 2016).

### Tyrosine Hydroxylase (TH) Immunostaining

The mice were anesthetized with chloral hydrate (400 mg/kg, i.p.), and then perfused with 4% paraformaldehyde. The free-floating sections (40 μm) were incubated with the antibody of chicken anti-TH (1:1000, Abcam) at 4°C overnight, and then in the antibody of biotin-labeled goat anti-chicken IgG antibody (1:500, Abcam) for 2 h at room temperature. Immunoreactivities were visualized using an avidin-biotin horseradish peroxidase complex (Vector Laboratories, Inc., Burlingame, CA, United States). TH-positive fibers were observed using a conventional light microscope (40× objective, DP70; Olympus).

### Statistical Analysis

The data were retrieved and processed with the software, Microcal Origin 9.1. The group data are expressed as the mean ± standard error (SE). Experimental results were compared among treatment groups by ANOVAs followed by Bonferroni’s *post hoc* test. Statistical analyses were performed using Stata7 software (STATA Corporation, United States). *p* < 0.05 was considered statistically significant.

## Results

### MPTP-Induced Dopamine Depletion *via* Decrease of D2R Activation Enhances Glutamate Release in BLA

**FIGURE 1:**
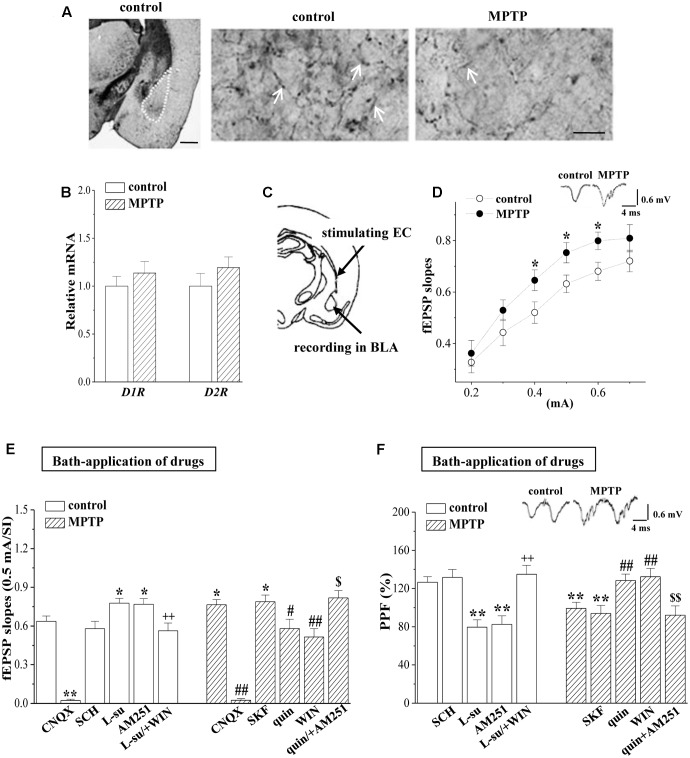
MPTP-induced dopamine depletion enhances presynaptic glutamate release. **(A)** Representative photomicrograph of TH-immunohistochemical staining in control mice (control) and MPTP-mice (MPTP). The BLA area is indicated by a white broken line (left panel). Scale bars = 1 mm. The arrows indicate TH positive fibers in BLA at high magnification. Scale bars = 50 μm. **(B)** Bar graphs show levels of *D1R* and *D2R* mRNA in BLA of control mice and MPTP-mice. **(C)** Schematic illustrating the locations of stimulating external capsule (EC) fibers and field potential recording in BLA. **(D)** Input–output (I/O) curve in BLA. Each point represents the group mean of the fEPSP slopes against stimulating intensities (SIs) from 0.2 to 0.7 mA. Two-way ANOVA, MPTP: *F*_(1,84)_ = 15.393, *p* < 0.001; SI: *F*_(5,84)_ = 29.953, *p* < 0.001; MPTP × SI: *F*_(5,84)_ = 0.325, *p* < 0.001. ^∗^*p* < 0.05 vs. control mice. **(E,F)** Bar graphs show the mean of fEPSP slopes (0.5 mA/SI) or PPF (75 ms IPI, %) in the slices of control mice treated with D1R antagonist SCH23390 (SCH), D2R antagonist L-sulpiride (L-su), CB1R antagonist AM251, AMPA receptor antagonist CNQX, or the co-application of L-sulpiride and CB1R agonist WIN55,212-2 (L-su/+WIN); in the slices of MPTP-mice treated with D1R agonist SKF38393 (SKF), D2R agonist quinpirole (quin), CB1R agonist WIN55,212-2 (WIN), AMPA receptor antagonist CNQX or the co-application of quinpirole and AM251 (quin/+AM251). ^∗^*p* < 0.05 and ^∗∗^*p* < 0.01 vs. control mice; ++*p* < 0.01 vs. control mice treated with L-su; ^#^*p* < 0.05 and ^##^*p* < 0.01 vs. MPTP-mice; ^$^*p* < 0.05 and ^$$^*p* < 0.01 vs. MPTP-mice treated with quin.

Consistent with MPTP-induced reduction in the tyrosine hydroxylase (TH) positive cells in VTA and SNpc (Zhang et al., 2016), the density of TH positive fibers in BLA was significantly reduced by MPTP-injection for 5 days in mice (MPTP-mice) (**Figure 1A**). Dopamine depletion by MPTP in caudate and putamen has been reported to increase the densities D1R and D2R (Rico et al., 2017). Although the MPTP-mice had a tendency to elevate the levels of *D1R* and *D2R* mRNA in BLA, the group when compared with control mice failed to reach the significance (*p* > 0.05, *n* = 6 mice; **Figure 1B**).

By stimulating the EC fibers (**Figure 1C**), we recorded a fEPSP in the BLA using a field potential recording, termed “EC-BLA synaptic transmission”. An input–output curve was created by plotting fEPSP slopes against stimulating intensities (SIs) from 0.2 to 0.7 mA. As shown in **Figure 1D**, the fEPSP slopes (0.4–0.6 mA/SI) in MPTP-mice were significantly increased compared to controls (*p* < 0.05, *n* = 8 slices/6 mice). Either basal fEPSP slopes in control mice or the increased fEPSP slopes in MPTP-mice were abolished by the addition of 10 μM AMPA receptor antagonist CNQX (*p* < 0.01, *n* = 8 slices/6 mice; **Figure 1E**). The PPF of fEPSP slopes was induced by delivering paired-pulse stimulation (0.5-mA/SI) with a 75 ms IPI to evaluate the probability of presynaptic glutamate release. The PPF value in MPTP-mice was less than that in control mice (*p* < 0.01, *n* = 8 slices/6 mice; **Figure 1F**). The increased fEPSP slopes or the reduced PPF value in MPTP-mice could be corrected by the bath-application of the D2R agonist quinpirole (10 μM, fEPSP slopes: *p* < 0.05, *n* = 8 slices/6 mice; PPF value: *p* < 0.01, *n* = 8 slices/6 mice) or the CB1R agonist WIN55,212-2 (10 μM, fEPSP slopes: *p* < 0.01, *n* = 8 slices/6 mice; PPF value: *p* < 0.01, *n* = 8 slices/6 mice), but not the D1R agonist SKF38393 (10 μM, *p* > 0.05, *n* = 8 slices/6 mice). The effects of quinpirole on the increased fEPSP slopes or the reduced PPF value in MPTP-mice were blocked by the co-application of CB1R antagonist AM251 (10 μM) (fEPSP slopes: *p* < 0.05, *n* = 8 slices/6 mice; PPF value: *p* < 0.01, *n* = 8 slices/6 mice). In the slices of the control mice, the bath-application of the D2R antagonist L-sulpiride (20 μM) or AM251 (10 μM) caused an increase in the fEPSP slope (L-su: *p* < 0.05, *n* = 8 slices/6 mice; AM251: *p* < 0.05, *n* = 8 slices/6 mice) with a reduction of the PPF value (L-su: *p* < 0.01, *n* = 8 slices/6 mice; AM251: *p* < 0.01, *n* = 8 slices/6 mice), whereas the D1R antagonist SCH23390 (10 μM) did not (*p* > 0.05, *n* = 8 slices/6 mice). The effects of L-sulpiride on the fEPSP slope and the PPF value were sensitive to the co-application of WIN55,212-2 (fEPSP slopes: *p* < 0.01, *n* = 8 slices/6 mice; PPF value: *p* < 0.01, *n* = 8 slices/6 mice). The results indicate that the activation of D2R inhibits the glutamate release from EC fibers in a CB1R-dependent manner; the MPTP-induced dopamine depletion through reducing D2R and CB1R activation enhances the glutamate release.

### MPTP-Induced Dopamine Depletion Attenuates GABAergic Neurotransmission in BLA

**FIGURE 2:**
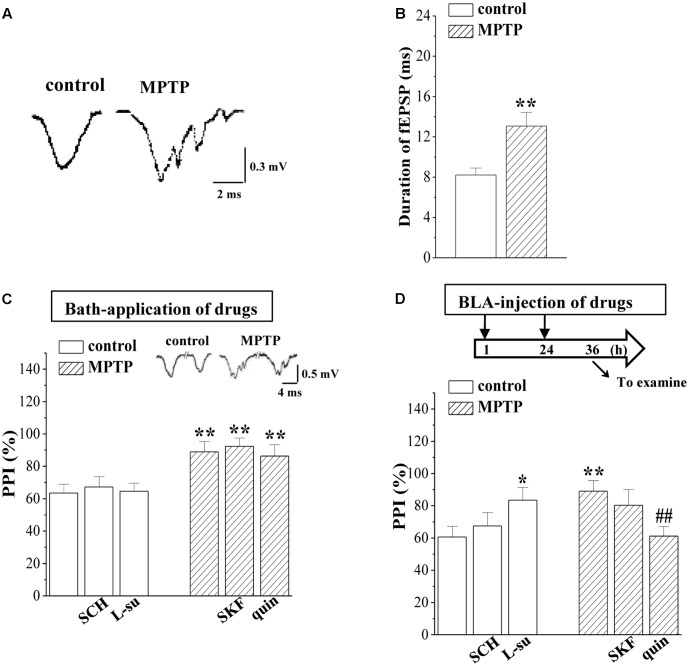
MPTP-induced dopamine depletion attenuates GABAergic neurotransmission. **(A)** Representative traces of fEPSP evoked by 0.5 mA/SI in control mice (control) and MPTP-mice (MPTP). **(B)** Bar graphs show the mean duration (ms) of fEPSP in control mice and MPTP-mice. ^∗∗^*p* < 0.01 vs. control mice. **(C,D)** Bar graphs show the PPI (25 ms IPI, %) in the slices of control mice treated with SCH23390 (SCH) or L-sulpiride (L-su) for 20 min; in the slices of MPTP-mice treated with SKF38393 (SKF) or quinpirole (quin); in control mice treated with BLA-injection of SCH23390 (SCH) or L-sulpiride (L-su) for 2 days; in MPTP-mice treated with BLA-injection of SKF38393 (SKF) or quinpirole (quin). **(D)** Time chart of the experimental procedure (upper panel). ↓: time of drug administration; ↘: time of electrophysiological experiments. ^∗^*p* < 0.05 and ^∗∗^*p* < 0.01 vs. control mice; ^##^*p* < 0.01 vs. MPTP-mice (two-way ANOVA).

A large body of evidence has established that abundant GABAergic local circuits in BLA build an excitation-inhibition cycle (Isoardi et al., 2004). The GABAergic inhibition is reduced in the dopamine-depleted animals (Janssen et al., 2016). Compared with the single waveform fEPSP in control mice, the same stimulation elicited a multi-spike waveform fEPSP in the BLA of MPTP-mice (**Figure 2A**). Additionally, the fEPSP duration in MPTP-mice was longer than that in control mice (*p* < 0.01, *n* = 8 slices/6 mice; **Figure 2B**). The PPI is considered to depend on GABAergic inhibitory circuit in BLA (Delaney and Sah, 2001). Notably, the PPI of fEPSP slopes induced by delivering paired-pulse stimulation (0.5 mA/SI) with 25 ms IPI in MPTP-mice was increased compared to that of the control mice (*p* < 0.01, *n* = 8 slices/6 mice; **Figure 2C**). The increased PPI value in MPTP-mice was insensitive to the bath-application of quinpirole or SKF38393 (*p* > 0.05, *n* = 8 slices/6 mice). However, the BLA-injection of quinpirole (0.5 μg/mouse) for continuous 2 days (**Figure 2D**) corrected the increased PPI value (*p* < 0.01, *n* = 8 slices/6 mice), but SKF38393 (0.1 μg/mouse) had no effects (*p* > 0.05, *n* = 8 slices/6 mice). The BLA-injection of L-sulpiride (0.25 μg/mouse) for 2 days increased the PPI value in control mice (*p* < 0.05, *n* = 8 slices/6 mice), whereas the administration of SCH23390 (0.25 μg/mouse) could not (*p* > 0.05, *n* = 8 slices/6 mice).

### MPTP-Induced Dopamine Depletion Causes GABA_A_R Down-Regulation in BLA

**FIGURE 3:**
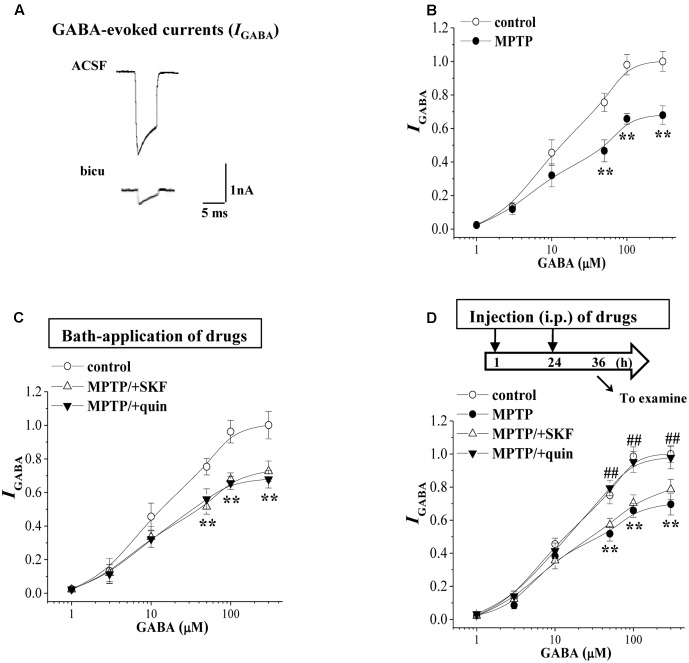
MPTP-induced dopamine depletion causes GABA_A_R dysfunction. **(A)** Representative traces of whole cell currents evoked by 10 μM GABA (*I*_GABA_) in control slices or the slices treated with bicuculline (bicu). **(B)** The densities of *I*_GABA_ evoked by GABA (1–300 μM) in control mice and MPTP-mice. Each point represents the density of *I*_GABA_ normalized by a control value evoked by GABA (10 μM). Repeated-measure ANOVA, *F*_(1,14)_ = 37.32, *p* < 0.001. ^∗∗^*p* < 0.01 vs. control mice. **(C)** Evoked *I*_GABA_ by GABA (1–300 μM) in slices of MPTP-mice treated with SKF38393 (SKF) or quinpirole (quin). ^∗∗^*p* < 0.01 vs. control mice. **(D)** Evoked *I*_GABA_ by GABA (1–300 μM) in MPTP-mice treated with the injection (i.p.) of SKF38393 (SKF) or quinpirole (quin). Time chart of the experimental procedure (upper panel). ↓: time of drug administration; ↘: time of electrophysiological experiments. Repeated-measure ANOVA, MPTP: *F*_(1,14)_ = 28.372, *p* < 0.001; SKF: *F*_(1,14)_ = 1.094, *p* = 0.313; quin: *F*_(1,14)_ = 32.941, *p* < 0.001. ^∗∗^*p* < 0.01 vs. control mice; ^##^*p* < 0.01 vs. MPTP-mice.

GABA can exert shunting effects on excitatory transmission mainly *via* GABA_A_R (Nishiyama et al., 2010). Further experiments were designed to examine the GABA_A_R activity in BLA principal neurons using a whole cell patch-clamp recording. Local perfusion with the GABA_A_R agonist GABA (10 μM) evoked an inward currents (*I*_GABA_) (**Figure 3A**), which was largely blocked by the GABA_A_R antagonist bicuculline (10 μM, *p* < 0.01, *n* = 5 cells/4 mice). With repeated applications of GABA in 5 min interval, the densities of *I*_GABA_ remained stable and exhibited no rundown for over 30 min (data not shown). The densities of *I*_GABA_ in MPTP-mice were less than those in control mice (*F*_1,14_ = 37.315, *p* < 0.001; **Figure 3B**). The EC50 value of the dose-response curve of *I*_GABA_ had no difference between MPTP mice (13.33 ± 1.07 μM) and control mice (12.57 ± 1.52 μM; *p* > 0.05). The bath-application of SKF38393 or quinpirole failed to alter the densities of *I*_GABA_ in MPTP-mice (*p* > 0.05, *n* = 8 cells/6 mice; **Figure 3C**). However, the injection (i.p.) of quinpirole (2 mg/kg) for 2 days (**Figure 3D**) could correct the reduced *I*_GABA_ in MPTP-mice (*p* < 0.01, *n* = 8 cells/6 mice), but SKF38393 (10 mg/kg) did not (*p* > 0.05, *n* = 8 cells/6 mice).

### MPTP-Induced Dopamine Depletion Suppresses GABA_A_R-α2 Expression

**FIGURE 4:**
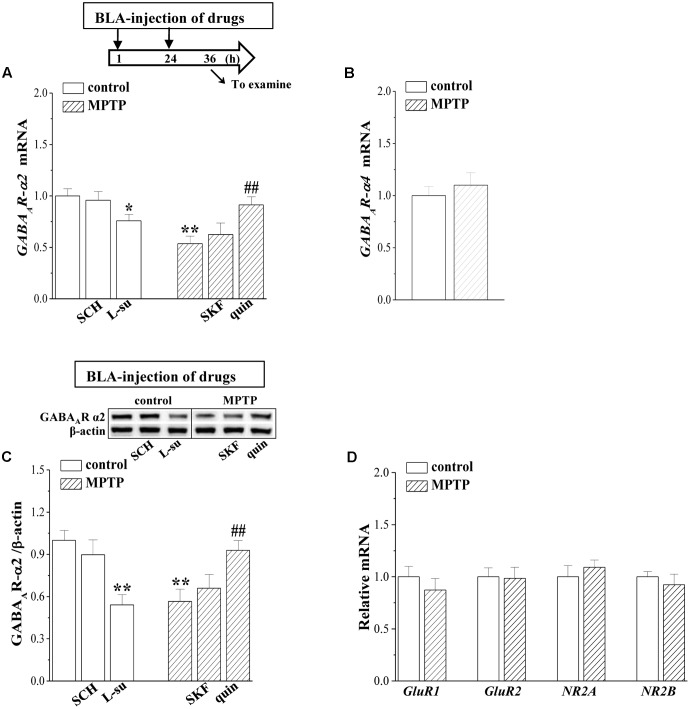
MPTP-induced dopamine depletion suppresses GABA_A_R expression. **(A–C)** Bar graphs show levels of *GABA_A_R-α2* and *GABA_A_R-α4* mRNA or GABA_A_R-α2 protein in BLA of control mice treated with BLA-injection of SCH23390 (SCH) or L-sulpiride (L-su); in MPTP-mice treated with BLA-injection of SKF38393 (SKF) or quinpirole (quin). Densitometric values of proteins normalized by the level of β-actin were normalized by control levels. **(A)** Time chart of the experimental procedure (upper panel). ↓: time of drug administration; ↘: time of RT-PCR and Western blotting. ^∗^*p* < 0.05 and ^∗∗^*p* < 0.01 vs. control mice; ^##^*p* < 0.01 vs. MPTP-mice (two-way ANOVA). **(D)** The levels of *GluR1*, *GluR2*, *NR2A*, *NR2B* mRNA in BLA of control mice and MPTP-mice.

The GABAergic interneuron expresses primarily α subunit-containing GABA_A_R (McDonald and Mascagni, 2004). BLA principal neurons contain primarily the α2 subunit (Marowsky et al., 2004); thus, we measured the GABA_A_R-α2 and GABA_A_R-α4 expression in BLA by RT-PCR and Western blotting, respectively. The level of *GABA_A_R-α2* mRNA in MPTP-mice was significantly reduced compared to that in control mice (*p* < 0.01, *n* = 6 mice; **Figure 4A**), but the levels of *GABA_A_R-α4* mRNA had no significant difference between control mice and MPTP-mice (*p* > 0.05, *n* = 6 mice; **Figure 4B**). The level of GABA_A_R-α2 protein in MPTP-mice was lower than that in control mice (*p* < 0.01, *n* = 6 mice; **Figure 4C**). The BLA-injection of quinpirole for continuous 2 days corrected the decreases in the levels of *GABA_A_R-α2* mRNA (*p* < 0.01, *n* = 6 mice) and protein (*p* < 0.01, *n* = 6 mice), but SKF38393 could not (*p* > 0.05, *n* = 6 mice). In control mice, the blockade of D2R by L-sulpiride caused a decrease in the *GABA_A_R-α2* mRNA (*p* < 0.05, *n* = 6 mice) and protein (*p* < 0.01, *n* = 6 mice), whereas the D1R antagonist SCH23390 failed to affect the levels of GABA_A_R-α2 expression (*p* > 0.05, *n* = 6 mice). In contrast, the levels of AMPA receptor (*GluR1* and *GluR2* mRNA) or NMDA receptor (*NR2A* and *NR2B* mRNA) expression in MPTP-mice did not significantly differ from those of control mice (*p* > 0.05, *n* = 6 mice; **Figure 4D**).

### MPTP-Reduced D2R-Mediated PKC Activity Suppresses GABA_A_R Expression

**FIGURE 5:**
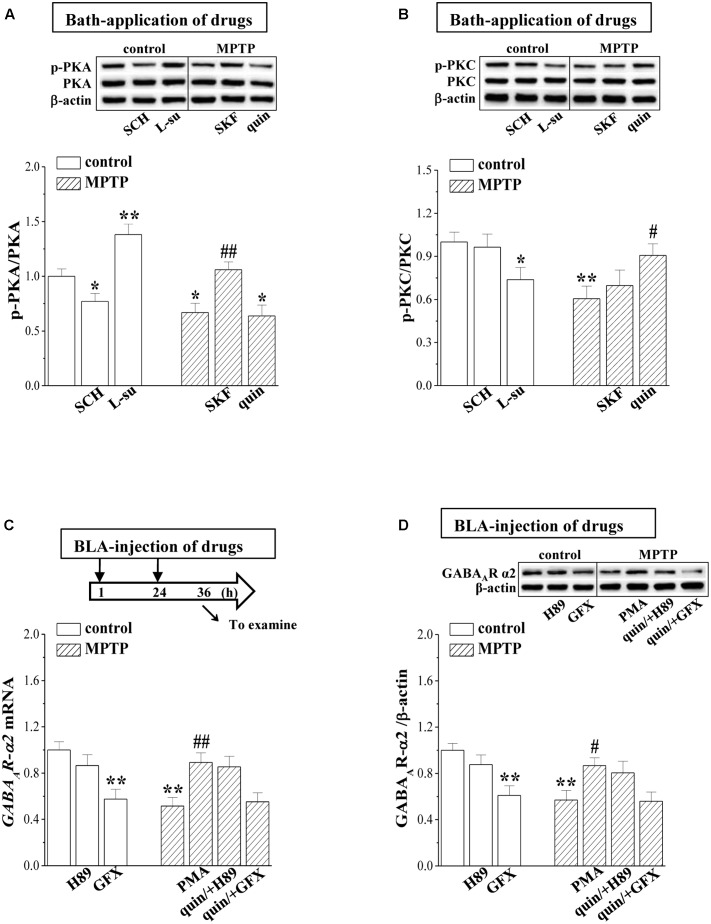
MPTP-reduced PKC signaling suppresses GABA_A_R expression. **(A,B)** Representative blots of phospho-PKA and phospho-PKC in BLA of control mice treated with bath-application of SCH23390 (SCH) or L-sulpiride (L-su) and in MPTP-mice treated with bath-application of SKF38393 (SKF) or quinpirole (quin). Densitometric values of phospho-PKA and phospho-PKC normalized by the PKA and PKC protein were normalized by control levels. ^∗^*p* < 0.05 and ^∗∗^*p* < 0.01 vs. control mice; ^#^*p* < 0.05 and ^##^*p* < 0.01 vs. MPTP-mice (two-way ANOVA). **(C,D)** Bar graphs show levels of *GABA_A_R-α2* mRNA and GABA_A_R-α2 protein in BLA of control mice treated with BLA-injection of H89 or GF109203X (GFX); in MPTP-mice treated with BLA-injection of PMA, or the co-administration of quinpirole and H89 (quin/+H89) or GF109203X (quin/+GFX). **(C)** Time chart of the experimental procedure (upper panel). ↓: time of drug administration; ↘: time of RT-PCR and Western blotting. ^∗^*p* < 0.05 and ^∗∗^*p* < 0.01 vs. control mice; ^#^*p* < 0.05 and ^##^*p* < 0.01 vs. MPTP-mice (two-way ANOVA).

D2R activation can decrease the phosphorylation of PKA (phospho-PKA; Yamaguchi et al., 2015), whereas it increases the PKC phosphorylation (phospho-PKC; Hong S.I. et al., 2016). The level of phospho-PKA in control mice was elevated by the bath-application of L-sulpiride for 20 min (*p* < 0.01, *n* = 6 mice; **Figure 5A**) and was reduced by SCH23390 (*p* < 0.05, *n* = 6 mice). The level of phospho-PKA in MPTP-mice was lower than that in control mice (*p* < 0.05, *n* = 6 mice), which was rescued by SKF38393 (*p* < 0.01, *n* = 6 mice) rather than quinpirole (*p* > 0.05, *n* = 6 mice). In comparison with control mice, the level of phospho-PKC was reduced in MPTP-mice (*p* < 0.01, *n* = 6 mice; **Figure 5B**), which was corrected by the bath-application of quinpirole (*p* < 0.05, *n* = 6 mice) but not SKF38393 (*p* > 0.05, *n* = 6 mice). The application of L-sulpiride decreased the phospho-PKC in control mice (*p* < 0.05, *n* = 6 mice), whereas SKF38393 failed to alter the level of phospho-PKC (*p* > 0.05, *n* = 6 mice). The levels of PKA and PKC proteins exhibited no significant difference between control mice and MPTP-mice (*p* > 0.05, *n* = 6 mice).

The PKA activation negatively regulated the GABA_A_R expression, while the PKC activation enhanced GABA_A_R expression (Bohnsack et al., 2016). In control mice, the BLA-injection of the PKC inhibitor GF109203X reduced the levels of *GABA_A_R-α2* mRNA (*p* < 0.01, *n* = 6 mice; **Figure 5C**) and protein (*p* < 0.01, *n* = 6 mice; **Figure 5D**). The BLA-injection of the PKC activator PMA in MPTP-mice corrected the decreases in the levels of *GABA_A_R-α2* mRNA (*p* < 0.01, *n* = 6 mice) and protein (*p* < 0.05, *n* = 6 mice). Furthermore, the BLA-injection of GF109203X could prevent the quinpirole-corrected GABA_A_R expression in MPTP-mice (*p* < 0.01, *n* = 6 mice). By contrast, the PKA inhibitor H89 did not cause changes in the level of GABA_A_R-α2 expression in control mice (*p* > 0.05, *n* = 6 mice).

### MPTP-Reduced GABA_A_R Activity Impairs LTD Induction

**FIGURE 6:**
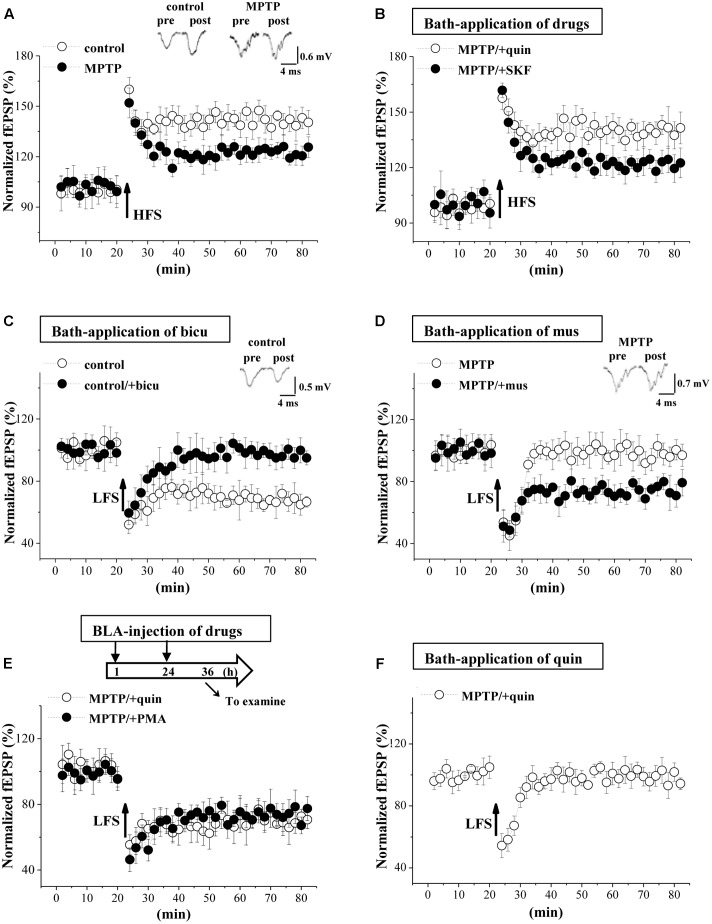
MPTP-reduced GABA_A_R activity impairs LTD induction. **(A)** LTP induction by applying high-frequency stimulation (HFS) in control mice and MPTP-mice. Each point represents the mean value of fEPSP slopes expressed as a percentage of baseline. A solid arrow indicates when HFS was given. **(B)** LTP induction in slices of MPTP-mice treated with bath-application of SKF38393 (SKF) or quinpirole (quin). **(C)** LTD induction by low-frequency stimulation (LFS) in slices of control mice treated with bicuculline (bicu). **(D)** LTD induction in slices of MPTP-mice treated with muscimol (mus). **(E)** LTD induction in MPTP-mice treated with BLA-injection (BLA) of SKF38393 (SKF) or quinpirole (quin). Time chart of the experimental procedure (upper panel). ↓: time of drug administration; ↘: time of electrophysiological experiments. **(F)** LTD induction in slices of MPTP-mice treated with bath-application of quinpirole (quin).

GABAergic disinhibition has been reported to affect synaptic plasticity in BLA (Rodriguez Manzanares et al., 2005). To test this possibility, we further examined the frequency-dependent LTP and LTD induction at EC-BLA synaptic transmission in control mice and MPTP-mice (*n* = 6 slices/6 mice). By delivering high-frequency (100 Hz) stimulation (HFS) in control mice, the fEPSP slopes were increased by approximately 40% for over 60 min (**Figure 6A**), indicative of LTP induction. A decrease in the amplitude of LTP was observed in MPTP mice compared to control mice (*p* < 0.05, *n* = 6 slices/6 mice; at 55–60 min after HFS), which could be recovered by the bath-application of quinpirole (*p* < 0.05, *n* = 6 slices/6 mice) rather than SKF38393 (*p* > 0.05, *n* = 6 slices/6 mice; **Figure 6B**). Additionally, the fEPSP slopes were reduced by low-frequency (1 Hz) stimulation (LFS) at 900 pulses in control mice (*n* = 6 slices/6 mice; **Figure 6C**), indicative of LTD induction, which was blocked by the GABA_A_R antagonist bicuculline (*n* = 6 slices/6 mice). The same LFS protocol could not induce the production of LTD in MPTP-mice (*n* = 6 slices/6 mice; **Figure 6D**). The bath-application of the GABA_A_R agonist muscimol recovered the LTD induction in MPTP-mice. Additionally, the BLA-injection of quinpirole or PMA for 2 days (**Figure 6E**) in MPTP-mice could rescue the LTD induction (*n* = 6 slices/6 mice). In contrast, the bath-application of quinpirole failed to recover the LTD induction in MPTP-mice (*n* = 6 slices/6 mice; **Figure 6F**).

### Involvement of MPTP-Impaired LTD Induction in Affective Disorder

**FIGURE 7:**
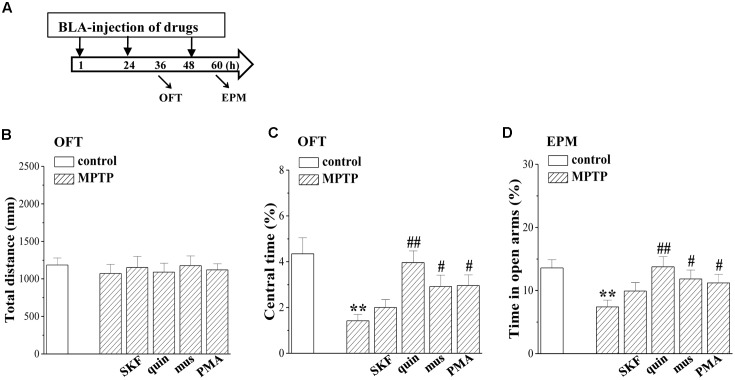
Involvement of MPTP- impaired LTD induction in affective disorder. **(A)** Time chart of the experimental procedure. ↓: time of drug administration; ↘: time of OFT and EPM, respectively. **(B–D)** Bar graphs show the distance traveled in OFT, the time spent in the central partition, and the time in open arms of EPM in control mice and MPTP-mice treated with BLA-injection of muscimol (mus), SKF38393 (SKF), quinpirole (quin) or PMA. Two-way ANOVA, **(C)** MPTP: *F*_(1,69)_ = 16.534, *p* < 0.001; drugs: *F*_(1,69)_ = 22.817, *p* = 0.008, **(D)** MPTP: *F*_(1,69)_ = 9.881, *p* = 0.002; drugs: *F*_(1,69)_ = 7.596, *p* = 0.007. ^∗∗^*p* < 0.01 vs. control mice; ^#^*p* < 0.05 and ^##^*p* < 0.01 vs. MPTP-mice.

To explore the relation of the altered EC-BLA synaptic LTP and LTD to anxiety-like behaviors, an open-field test (OFT) and elevated plus-maze (EPM) were conducted on days 3–4 after administration of MPTP and drugs (**Figure 7A**). The distance traveled in the OFT did not differ between control mice and MPTP-mice (*p* > 0.05, *n* = 12 mice; **Figure 7B**), but MPTP-mice spent less time in the central partition of the arena than control mice (*p* < 0.01, *n* = 12 mice; **Figure 7C**). Additionally, the time in the open arms of the EPM was reduced in MPTP-mice compared to control mice (*p* < 0.01, *n* = 12 mice; **Figure 7D**). The BLA-injection of muscimol (*p* < 0.05, *n* = 12 mice), quinpirole (*p* < 0.01, *n* = 12 mice) or PMA (*p* < 0.05, *n* = 12 mice) in MPTP-mice could correct the time spent in the central partition of OFT and open arms of EPM, but SKF38393 did not (*p* > 0.05, *n* = 12 mice).

## Discussion

**FIGURE 8:**
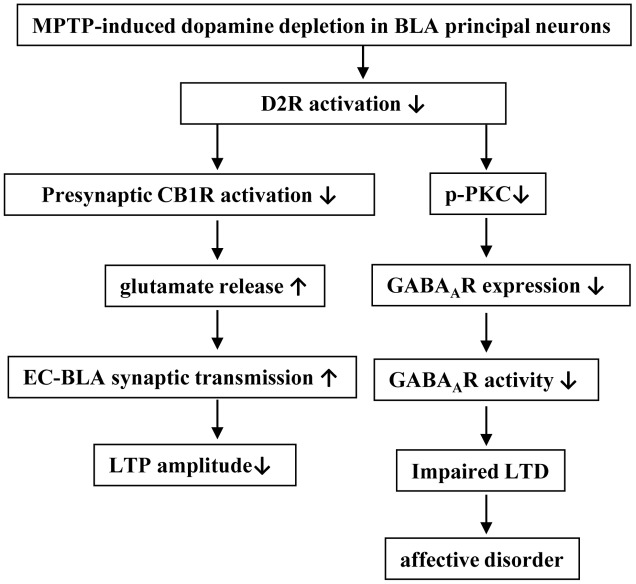
The hypothesis of molecular mechanisms underlying the MPTP-reduced dopaminergic afferent in BLA to induce affective disorder. ↑: increase; ↓: decrease.

Using field potential recording and patch-clamp recording, and by combined pharmacological experiments, our results indicate that the MPTP-induced dopamine depletion in BLA through reducing D2R activation enhanced the presynaptic glutamate release at the EC-BLA synaptic transmission and decreased the LTP amplitude; the MPTP-induced dopamine depletion *via* the decrease of D2R activation suppressed the expression and function of GABA_A_R in the BLA principal neurons to reduce GABA_A_R-mediated inhibition, which impaired the LTD induction, leading to the production of anxiety-like behaviors (**Figure 8**).

A principal observation in this study is that the densities of *I*_GABA_ were reduced in BLA principal neurons of MPTP-mice. The activation of D1R has been reported to enhance the GABA_A_R mediated IPSCs, whereas the activation of D2R depresses GABA_A_R activity (Trantham-Davidson et al., 2004). Li et al. (2011) reported that D2R activation facilitates GABA_A_R insertion at newly formed inhibitory synapses. However, the decreased density of *I*_GABA_ in MPTP-mice failed to be altered by the bath-application of D2R or D1R agonist. By contrast, the administration of the D2R agonist for 2 days in MPTP mice was able to correct the decreased *I*_GABA_ density. Consistently, the level of GABA_A_R-α2 expression in BLA was significantly reduced in MPTP mice, which could be rescued by the D2R agonist. In mice lacking D2R, the GABAergic neurotransmission is reduced although the level of glutamic acid decarboxylase (GAD) is strongly increased (An et al., 2004). Chronic D2R stimulation has been reported to increase the density of post-synaptic GABA_A_R clusters (Lalchandani et al., 2013). Therefore, it is highly likely that the MPTP-induced dopamine depletion *via* the decrease of D2R activation in the BLA principal neurons suppresses GABA_A_R expression leading to the dysfunction of GABA_A_R.

The levels of phospho-PKA and phospho-PKC in the BLA of MPTP mice were lower than those in control mice. The D1R agonist could recover the level of phospho-PKA in MPTP mice, and the D1R antagonist caused the decline of phospho-PKA in control mice. In MPTP mice, the decreased phospho-PKC was rescued by the activation of D2R rather than D1R. In control mice, the blockade of D2R inhibited the phospho-PKC and elevated the phospho-PKA. The PKC activation increases GABA_A_R-α4 expression, while the PKA activation decreases synaptic GABA_A_R-α4 expression (Bohnsack et al., 2016). PKC activation can phosphorylate the GABA_A_R subunits at α4 S443 (Nakamura et al., 2015) to increase surface expression of GABA_A_R-α4 (Abramian et al., 2010; Werner et al., 2011). The activation of D2R selectively up-regulates the GABA_A_R expression probably *via* PKA signaling (Pan et al., 2008). However, the level of GABA_A_R-α4 expression in MPTP mice did not differ significantly from control mice. The BLA principal neurons primarily express the α2 subunit (Marowsky et al., 2004). Interestingly, the decreases in the *GABA_A_R-α2* mRNA and protein in MPTP mice were corrected by the PKC activator. The inhibition of PKC rather than PKA reduced the GABA_A_R-α2 expression in control mice. Moreover, the PKC inhibitor abolished the protective effects of D2R agonist on MPTP-suppressed GABA_A_R-α2 expression, but the PKA inhibitor did not. The results give an indication that the MPTP-induced dopamine depletion *via* the down-regulation of D2R-mediated PKC signaling suppresses GABA_A_R-α2 expression.

The MPTP-induced dopamine depletion destructed the balance between excitatory and inhibitory circuits in BLA. Flores-Hernandez et al. (1997) reported that the transmission of EC-BLA glutamatergic synapses is potentiated by activation of D1R or inhibition of D2R. In the slices obtained from control mice, the application of D2R or CB1R antagonist was able to increase the fEPSP slopes with decrease in the PPF value. And also, the effects of D2R antagonist on the fEPSP slopes and PPF value were blocked by the CB1R agonist. The results indicate that the block of D2R in BLA principal neurons through reducing release of post-synaptic endocannabinoids causes the inactivation of presynaptic CB1R to enhance the glutamate release. In MPTP-mice, the EC-BLA synaptic efficiency was enhanced with the reduction of PPF, which was corrected by the activation of D2R or CB1R rather than D1R. In particular, the effect of D2R agonist on glutamate release in MPTP-mice was abolished by the blockade of CB1R. Thus, it is conceivable that the MPTP-induced dopamine depletion *via* the decrease of D2R activation leads to the decline of post-synaptic endocannabinoids release, which enhances presynaptic glutamate release to potentiate the EC-BLA excitatory. On the other hand, a multi-spike waveform fEPSP was observed in MPTP-mice with prolonged fEPSP duration and increased PPI value, reflecting a deficit in the GABAergic inhibition circuit (Delaney and Sah, 2001). The activation of presynaptic CB1R has been reported to inhibit the GABA release (Foldy et al., 2006). Interestingly, the BLA-injection of the D2R agonist for 2 days could recover the GABA_A_R-mediated inhibition, but the bath-application of the D2R agonist had no effects. Therefore, it is indicated that the MPTP-induced dopamine depletion *via* the decrease of D2R activation reduces the GABA_A_R expression rather than the GABA release leading to the deficits in the BLA inhibitory circuits.

The LTP induction at BLA glutamatergic synapses depends on the Ca^2+^ influx from the NMDA receptor (Mirante et al., 2014). A possible explanation for the decreased amplitude of LTP in MPTP-mice is that the increased glutamate release causes NMDA receptor activation prior to LTP induction, which elicits presynaptic inhibition when LTP is induced through releasing retrograde factor(s) (Kato et al., 1999). This idea is supported by our experimental results that the bath-application of D2R agonist recovered the normal LTP amplitude in MPTP-mice. Additionally, the PKA signaling is involved in the induction of LTP at the cortical input to the lateral amygdala synapses (Fourcaudot et al., 2008). Although the D1R agonist recovered the phospho-PKA in MPTP-mice, it did not correct the LTP amplitude. The GABAergic inhibitory circuit in BLA is very important for the LTD induction (Rammes et al., 2001) and controlling motion and attention behavior (Martijena et al., 2002). The GABAergic system in the amygdala networks appears to be involved in generating responses to fear-conditioned stimuli (Ehrlich et al., 2009) and is crucial for processes of fear extinction learning (Sangha et al., 2009). The impairment of LTD in MPTP-mice was rescued by the bath-application of the GABA_A_R agonist or the BLA-injection of the D2R agonist or the PKC activator. Consistently, the anxiety-like behaviors in MPTP-mice were improved by the BLA-injection of the GABA_A_R and D2R agonists or PKC activator. Thus, the MPTP-impaired LTD induction in the BLA is likely responsible for the production of anxiety-like behaviors.

The results from the present study indicate that the MPTP-induced dopamine depletion *via* the decrease of D2R activation in BLA principal neurons suppresses GABA_A_R expression and activity, which impairs LTD induction leading to the production of anxiety-like behaviors (**Figure 8**). The present study provides insight into the mechanisms underlying anxiety disorders commonly observed in PD patients and potential therapeutic targets.

## Author Contributions

TZ and PC performed the field potential recording, immunostaining, western blotting, and all statistical analysis. TC and BZ undertook the whole cell patch-clamp recording and RT-PCR analysis. JH carried out the animal care and behavioral examination. LC designed the experiment and finished the manuscript.

## Conflict of Interest Statement

The authors declare that the research was conducted in the absence of any commercial or financial relationships that could be construed as a potential conflict of interest.
